# The Need for Sexuality Education in Pakistan

**DOI:** 10.7759/cureus.2693

**Published:** 2018-05-28

**Authors:** Asim Shaikh, Rohan Kumar Ochani

**Affiliations:** 1 Dow Medical College, Dow University of Health Sciences (DUHS), Karachi, Pakistan

**Keywords:** sexuality education, abortions, hiv, sexually transmitted illnesses

## Abstract

Sexuality education is an extremely controversial topic in countries like Pakistan where any dialogue regarding sexual practices is considered taboo. Yet, it is a country with a high prevalence of abortions and sexually transmitted illnesses (STIs). Current knowledge about such issues in the general public is also very poor. Hence, it is extremely important to discuss the advantages of having sexual education programs in Pakistan and analyze the current impact of an absence of such programs and public awareness campaigns.

## Editorial

Sexuality education has always been a topic of controversy, even in the developed world. Yet, most people around the globe agree that there has to be some form of sexuality education, in schools or as public awareness campaigns, to protect young individuals from avoidable pregnancies, sexually transmitted illnesses (STIs), and to impart knowledge on normal sexual behaviors in teenagers. However, in countries like Pakistan, where impositions on any sort of dialogue are strongly influenced by religious and traditional practices, public dialogues focusing on sexuality education are considered extremely taboo and invite great criticism and outrage from the public. Therefore, it is extremely important to outline the disadvantages stemming from the absence of such awareness and its implication on public health, especially on young individuals.

A study was conducted in Pakistan to outline the current misconceptions of men towards sexual practices. An overwhelming 94% of the respondents agreed to have masturbated yet 31.4% of them believed it caused physical illnesses and there was a high prevalence (76%) of its association with guilt [1]. Such misconceptions most likely arise from the fact that no form of sexuality education is provided in public schools in Pakistan.

Furthermore, a study estimated that in 2012, there were a total of 2.2 million abortions in Pakistan and that currently, the annual national abortion rate stands at 50 per 1000 women [2]. In a country where abortion is an extremely taboo topic, regardless of the reason, and is currently illegal if done for socio-economic reasons, these staggering rates suggest the dire need for public awareness campaigns, or at least some form of public discussion regarding safe sex practices, especially the use of contraceptive devices and condoms. The Joint United Nations Programme on HIV/AIDS (UNAIDS) estimates that in 2016 there were 130,000 people living with the human immunodeficiency virus (HIV) in Pakistan [3] and another study [4] found that amongst individuals living with STIs, knowledge about these illnesses is extremely poor. These findings reiterate the importance of increasing public knowledge regarding sexual practices as in spite of strong religious and traditional views on sexual intercourse, these illnesses continue to persist; imposing legal restrictions based on religious and cultural views have not helped in their decline. This is supported by a study conducted in the United States which found that overall, increased sexuality education within school criteria lowered adolescent birth rates; however, in states with high religiosity rankings and conservatism, adolescent birth rates tended to be higher [5]. The importance of sexuality education becomes even more important when it is considered that Pakistan, currently a country of more than 190 million people, suffers great resource shortages such as food and electricity, and cannot adequately sustain a high population growth rate.

The impact of having sexuality education cannot be overstated, especially in a country like Pakistan, where it can help tackle multiple problems at once such as extremely high population growth rates, STI contraction, and high abortion rates which are carried out by unlicensed, illegal abortion clinics.
